# Reproductive Output Reveals the Maternal Effects on Offspring Size-Number Trade-Off in Cultured Asian Yellow Pond Turtle (*Mauremys mutica*)

**DOI:** 10.3390/ani13132219

**Published:** 2023-07-06

**Authors:** Yakun Wang, Xiaoyou Hong, Xiaoli Liu, Wei Li, Chen Chen, Junxian Zhu, Chengqing Wei, Xinping Zhu, Lingyun Yu

**Affiliations:** Key Laboratory of Tropical & Subtropical Fishery Resource Application & Cultivation of Ministry of Agriculture and Rural Affairs, Pearl River Fisheries Research Institute, Chinese Academy of Fishery Sciences, Guangzhou 510380, China; wykzkyky@163.com (Y.W.);

**Keywords:** age, maternal size, reproductive strategies, size-number trade-off, cultivation management

## Abstract

**Simple Summary:**

We explored the effect of maternal size on offspring size and number using a 4-year dataset based on paternity analyses in Asian yellow pond turtle, *Mauremys mutica*, and found that the maternal size significantly affected the offspring number but not offspring size. The offspring size was significantly correlated with maternal age. Our results suggest that the offspring size-number trade-off theory does not apply to cultured *M. mutica* and also provides basic data for the efficient artificial breeding management of *M. mutica* in captivity.

**Abstract:**

Offspring size-number trade-off is a critical component of life-history theory and is important for further understanding the reproductive strategies of animals. The relationship between this trade-off and maternal size has been explored in several turtle species, except for the Asian yellow pond turtle, *Mauremys mutica*. To investigate how the maternal condition affects offspring size and number, we explored the relationships among the maternal body size and the number and size of cultured *M. mutica* hatchlings using a 4-year dataset. Our results showed that different females not only produced different sizes of offspring but also produced different numbers of offspring. No trade-off in egg size number was detected. According to regression analysis, we did not find that the maternal body size significantly influenced the offspring mass; however, we detected that the offspring size was significantly correlated with the clutch size and maternal age. The mean body mass of offspring increased with maternal age, and the clutch size varied significantly over four years, which was correlated with offspring size, maternal body size and age. However, the number of offspring per female increased with the maternal plastron length rather than age. Our results were inconsistent with the optimal offspring size theory in that females did not increase their offspring size but rather increased the offspring number to increase their fitness, which will also provide a basis for the efficient cultivation management of turtles.

## 1. Introduction

The offspring size and number are critical components in life-history traits and have notable effects on survival and population growth [1]. In a given environment, the available resources are allocated not only to growth, migration and defense but also for storage to improve the parents’ survival; therefore, the resources (i.e., food and space) available for reproduction are limited and usually lead to a trade-off between offspring size and number for the maternal reproductive investment [2,3,4]. The trade-off between the offspring size and number is adjusted in response to the unpredictable, fluctuating environmental conditions the progeny may encounter, which will improve fitness [5,6]. For instance, a female can produce fewer but larger progeny to increase their survival, thereby optimizing the offspring size when available resources are finite [7]. It has been shown that an improvement in offspring survival is associated with offspring size [8] and is dependent on the resources available and maternal fecundity [9]. For example, female leatherback turtles, *Dermochelys coriacea*, possess high reproductive output, laying the largest eggs per reproductive season to improve survival and to compensate for high and unpredictable mortality rates in early life-history stages [9].

There was a general consensus that large mothers produce larger offspring [10,11], and several hypotheses can explain the effects of maternal size on the offspring size and number: (1) sibling competition, induced by high-fecundity mothers, can allow bigger siblings to live through difficult periods, especially when there is little food or water and more predators, while smaller individuals died and were eliminated [12]; (2) maternal behaviors, such as nest choice, parental care and prevention of predation (e.g., the bigger female, the stronger ability in predation and prevention of predation), would provide a safe and comfortable environment and enough nutrition for the neonates [6]; (3) morphological constraints (e.g., different pelvic aperture widths and caudal gap heights attributed to different female sizes) on the offspring size and reproductive effort for turtles that have small clutches result in a divergence from the optimal egg size theory [10,13,14,15]; and (4) there is a trade-off between the current and subsequent reproductive strategy, especially for long-lived oviparous reptiles, as females tend to have a decreased offspring size in their next reproduction period when they encounter higher risk if they are producing larger eggs currently [16]. However, it is unclear whether the maternal size can influence the offspring size and number in yellow pond turtles, *Mauremys mutica*.

In China, *Mauremys mutica* is one of the most heavily harvested and traded turtle species [17], and therefore plays an important role in food consumption, ornamentation and aquaculture, with great economic and scientific research value. As a long-lived animal, *M. mutica* can lay eggs for many years, even though we do not know exactly how many years this has occurred so far in cultured conditions, as is the case for most long-lived species. However, there is rare work reported about the variations in egg laying among years in *M. mutica*; therefore, it is necessary and feasible to understand the breeding strategies under artificial cultivation conditions. The relationship between egg size and maternal body size has been reported in other turtles [10,18]. Larger females tend to produce larger clutches of heavier eggs, and the offspring will perform better (i.e., stronger locomotivity) than the offspring of smaller females [19]. A previous study reported the maternal size effect on the reproductive parameters of the *M*. *mutica* population [20]. However, there is no study on both individual and population levels reporting the relationship between maternal size and offspring size and number. Moreover, the vast majority of the studies about the relationship between the maternal size and offspring size-number trade-off were conducted in wild animals in natural conditions but not in farm conditions, where, after all, food is in ample supply. It is necessary and meaningful to explore whether the theory is suitable for reptiles cultured under artificial farming conditions from another perspective.

The present study focused on (1) comparing and analyzing the reproductive outputs among and within four consecutive years and between the consecutive four years within and among female Asian yellow pond turtles and (2) testing the hypotheses of the optimal offspring size theory by exploring the effects of maternal size and age on the offspring size and number in cultured *M. mutica* based on the parentage analysis for the mother turtles and their offspring.

## 2. Material and Methods

### 2.1. Experimental Animals

In our study, 84 female *M. mutica* were randomly selected from approximately 400 individuals born in the same month in 2005, and they were the F1 generation of a population (876 mature individuals) captured in Qinzhou (Guangxi Province, China) in 1998 [21]. They reached sexual maturity and laid eggs in May 2010. By 2013, they had been raised for 8 years in the turtle breeding farm of the Pearl River Fisheries Research Institute, Chinese Academy of Fishery Sciences, Guangzhou (23°03′50″ N, 113°13′11″ E). In order to ensure the stability of the external environment and to reduce the influence of other factors on reproduction, we continuously provided 5% of the total body weight of artificial compound feed daily and exchanged the water under aeration every three days for these individuals. Over four years of the experiment (2013–2016), from April to November of each year, we maintained 126 individuals (84 females and 42 males) in the same outdoor tank (26.32 m^2^ total area) containing 16.45 m^2^ of water area and 9.87 m^2^ of sandy area for nesting, whereas from December to March of each year, the mothers and their offspring were cultured in thermostatic chambers (the same size as the outdoor tank) at 25 °C with the lights set on an 11 L: 13D cycle (similar to the mean winter photoperiod at our study site).

### 2.2. Sample Collection

During the egg-laying season (April to August) from 2013 to 2016, we collected and registered the eggs and clutches, determined the fertilized eggs based on the white spot that appeared the following day, and incubated all fertilized eggs according to the protocol of a previous study in our laboratory [21]. Sediments stuck on the surface of fertilized eggs were removed with a soft brush prior to hatching. The fertilized eggs were placed in Styrofoam containers (80 × 40 × 20 cm, length × width × height) covered with vermiculite and incubated at a constant temperature of 29 °C, with a moderate amount of water sprayed on the vermiculite daily to ensure a constant humidity of 80%. The hatchlings were separated and reared in Styrofoam containers (220 × 180 × 120 mm, length × width × height) that were labeled with the records regarding their egg and clutch. The plastron lengths of the 84 female *M. mutica* were measured in March every year from 2013 to 2016 by a digital caliper to the nearest 0.01 cm. After the thorough absorption of the yolk sac, the hatchlings were marked and measured prior to feeding. The body mass of each neonate and the clutch mass were measured by electronic scale to the nearest 0.01 g. 

Genomic DNA was isolated from samples (nails) for the 84 female adults in 2013 and from all the neonates (including the dead hatchlings) produced from 2013 to 2016 with an OMEGA tissue kit following the manufacturer’s instructions. The genomic DNA was diluted to a concentration of 20 ng/μL prior to genotyping [22]. The reaction for each multiplex PCR assay contained 1 μL of genomic DNA, 5 μL of ABI Multiplex PCR Master Mix, 2 μL of primer, 0.2 μL of fluorescent labeled connectors, and double distilled water to a final volume of 10 μL. The qPCR conditions were as follows: 94 °C pre-denaturation for 5 min, 94 °C denaturation for 30 s, annealing for 40 s, extension at 72 °C for 40 s, 22 cycles; denaturation at 94 °C for 30 s, annealing at 53 °C for 40 s, extension at 72 °C for 30 s, 8 cycles, extension at 72 °C for 10 min. DNA amplification was performed by two multiplex PCR assays with 16 pairs of microsatellite loci [22], and then the amplification products for the samples were genotyped with capillary electrophoresis in the ABI3130 Genetic Analyser (Applied Biosystems, Inc., Carlsbad, CA, USA). 

### 2.3. Statistical Analyses

We performed two multiplex PCR systems with 16 pairs of microsatellites screened in our laboratory (see detailed information on the genotyping methods) [22] to conduct paternity analyses for the female turtles and their offspring. The relationships between the 4 generations of offspring collected over the four years and the 84 female turtles were verified by means of CERVUS 3.0 [23]. For the classification of paternity assignment, the default typing error rate is 1% at a 95% confidence level. During the egg-laying season, prenatal female body mass variation is ambiguous because eggs deposited in the vents partially account for the body mass, resulting in a vague relationship between female size and offspring size; therefore, the body mass is less reliable than the carapace length and plastron length [24]; thus, we chose the plastron length as the characteristic to assess the relationship between maternal body size and offspring size. 

To test for age-related or maternal size effects on the offspring size, we conducted repeated-measures ANOVA in a mixed model (linear) with a mean body mass of neonates per female every year as the dependent variable, where the year (maternal age) and maternal plastron length were fitted as fixed factors, and the maternal identity as a random factor [25]. A generalized linear model was used using negative binomial distribution for the annual number of offspring per female to investigate whether the offspring number is relevant to maternal size or age; the fixed factors were plastron size and age of the female, with the maternal identity as a random factor [26]. Data analysis was conducted in program R based on the data collected in our study, and a *p*-value of <0.05 represents the data being statistically significant.

The regression analysis was conducted between plastron length and the offspring characteristics based on the four years of data. The Shapiro–Wilk test was carried out to test for the normal distribution of the residuals. Pairwise Pearson’s correlation coefficients were analyzed to detect the linear correlations among these reproductive characteristics in GraphPad Prism, version 5.01, for Windows (GraphPad Software, San Diego, CA, USA).

Furthermore, to explore the trends of offspring size (mainly the body mass of the new hatchlings) and number (mainly the mean clutch size) over the four years, respectively, we calculated the mean body mass of the hatchlings and clutch size every year for the 84 females over the four consecutive years.

## 3. Results

### 3.1. Comparisons of the Reproductive Output among Four Years

A total of 1259 offspring from 633 clutches were calculated based on the parentage analyses of the 16 pairs of microsatellite loci (Table 1). The number of offspring produced by different females varied from 1 to 42 over the four years (mean ± SD, 14.92 ± 9.37). The egg-laying behavior of different females varies from year to year. Some females only laid eggs in one/two/three years, which was named “on-year or off-year egg-laying”, except in 2015, when all females laid eggs. There were significant differences detected in the offspring’s body mass but not in the number of offspring over the four consecutive years. Female size and age significantly affected the number and size of offspring, respectively (Table 2). 

### 3.2. Relationship between Maternal Size and Offspring Size and Number

The maternal plastron length showed a marked influence on the clutch size and clutch mass (Table 3, Figure S1A,B), as well as the number of clutches (*F*_1,82_ = 9.10, *r* = 0.32, *p* = 0.034). We also detected that the maternal plastron length significantly affected the total number of offspring (Table 3 and Figure S1C) but not the offspring’s body mass (Table 3). The clutch mass was significantly influenced by the mean offspring mass (hatchlings of the same clutch) (Figure S2A). We found a weak but significant positive relationship between the overall number of offspring and offspring body mass per female across the four years after normalizing the data with a log_10_ transformation (Figure S2B), and the mean offspring mass increased significantly with the clutch size (Figure S2C). The mean clutch size markedly influenced the overall number of offspring per female across four years (positive relationship) after normalization with a log_10_ transformation (Figure S2D).

### 3.3. Relationship between Maternal Age and Offspring Size-Number

**Figure 1:**
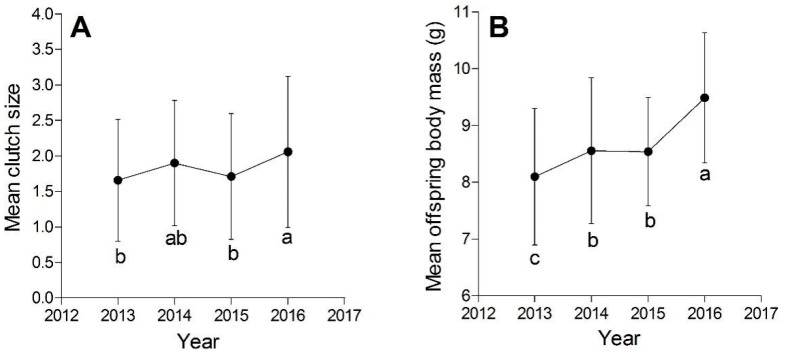
The mean number of offspring per clutch (mean clutch size) varied by year (**A**). Each point represents the mean clutch size of 84 females in each year. The mean body mass of offspring varied with the year (**B**). Each point represents the mean body mass of offspring produced by the 84 females in each year. Years not sharing the same lowercase letter are significantly different based on the post hoc analysis.

The number of offspring maintained constant yearly, without depending on the maternal age (*p* = 0.342), although the mean clutch size fluctuated markedly in some years (*F*_3,629_ = 5.82, *p* = 0.0006, Figure 1A). Moreover, the mean offspring body mass increased over the four years, and the maternal age influenced the offspring body mass significantly (Table 3 and Figure 1B). There was no interaction between the year and maternal plastron length on offspring body mass.

## 4. Discussion

In our study, the parentage analysis results revealed a marked difference in reproductive output among individuals, with the overall number of offspring ranging from 1 to 42 per female for the four-year period. Interestingly, some females laid more eggs in one year but laid fewer and even no eggs in the next year; this unstable phenomenon (on-year or off-year) was also found in other oviparous vertebrates, including *Chrysemys picta* [27], *Takydromus septentrionalis* [28] and *Emydocephalus annulatus* [29]. In cultured conditions, all the female turtles were farmed together in the same tank; therefore, the possible influence of stress or competition (e.g., food) due to the housing conditions may result in the different body sizes of females because of the differences in food ability (the bigger females, the greater predatory ability). Therefore, the significant differences in the number of offspring may generally result from the interaction between the maternal effects and environmental conditions [12]. In addition, Wallis et al. (1999) [30] determined that the annual egg production was a function of clutch size, which also supports our findings of significant differences in offspring numbers among years and a significant positive relationship between the mean clutch size and offspring number. We detected a significant relationship between the clutch frequency and the female plastron length during the four years. Accordingly, we assume that the ability of larger females to produce significantly more offspring may be correlated with a shorter inter-clutch interval and higher clutch frequency than that of small females because all the females shared the same egg production period in our work. This general tendency has also been found in lizards, which are small, short-lived species that produce small clutch sizes and multiple clutches [31]. Moreover, many species that have multiple clutches per year have variations in clutch size, e.g., one large clutch size is often followed by a smaller clutch size.

Previous studies have reported positive correlations between the hatchling size and maternal size in several species of freshwater turtles [32,33]. In this study population, we did not detect a significant positive relationship between the female body size and offspring size, which was similar to the result for *Chrysemys picta* [27]. Moreover, the offspring size was profoundly heritable in some birds (e.g., h2 = 60% in Ural owls; h, heritability) [25,34], and the offspring size plays a crucial role in increasing maternal fitness rather than increasing the offspring fitness [35].

Within species, the relationship between the number of offspring and offspring size may be masked because some females who have abundant resources (i.e., food) can invest more into reproduction, therefore producing larger and/or more offspring, while smaller mothers invest little or no energy into reproduction because of the scarcity of resources [36], and then produce smaller and/or fewer offspring. However, the relationship above occurs among individuals all the time [37]. Therefore, clarifying the reproductive allocation at the individual level enabled us to understand the evolution of the reproductive strategy.

As can be seen above, maternal body size, for *M. mutica*, influenced the number of offspring more significantly than it influenced the offspring size, implying that larger females invest more energy and nutrition into the offspring number than the offspring size in cultured conditions, while the subsequent result showed a significant positive correlation between the number of offspring and offspring size after we normalized the data with a log_10_ transformation. This means that large females produced not only more but also slightly larger offspring; however, there is no direct evidence of a significant correlation between maternal body size and offspring size. Our results differed from previous findings in turtles in that larger females tend to produce larger offspring [11,24], and these offspring are better able to survive challenges in adverse circumstances [38]. Therefore, our results documented that yellow pond turtles increased maternal fitness by producing more offspring first rather than larger offspring [9]. In other reptiles, such as sand lizards, *Lacerta agilis*, the offspring size is negatively correlated with the offspring number, which implies a diversity of offspring size-number trade-off effects among taxa [39]. In fact, we also found no evidence of a trade-off between the clutch size and offspring size, which was similar to the study in the red-banded wolf snakes, *Lycodon rufozonatus* [40].

Here, the mean body mass of newly hatched offspring every year increased over the four years, and there were significant differences found in the offspring body mass within the females across the years, except for 2014 and 2015. Given that the body masses of the neonates were not related to maternal size, we speculated that the offspring’s body mass might be strongly associated with maternal age [41], which is also verified in another research study of *M. mutica* [42]. It was reported that the age effect might have something to do with the “overhead” costs, which were ignored in previous studies and theory, in influencing reproduction and sexual maturation [43,44,45]. Overhead costs usually involve the development of the reproductive structure, especially during sexual maturation and maternal respiration when reproducing, as well as others. Therefore, the maternal reserve ultimately determines the reproductive output. It was well known that maternal size increased with age, and larger females suffered higher overhead costs of reproduction [44]. At the same time, costs that have a greater effect on the offspring size than on the offspring number will cause more variation in offspring size [44]. 

Additionally, we found that the mean number of offspring per clutch (mean clutch size) fluctuated annually, as did the offspring size, but not the number of offspring, which was constant with the result seen for the painted turtle, *Chrysemys picta*. They also showed the female’s age significantly indirectly influenced clutch sizes [46]. Unlike this, previous studies showed that the clutch size tended to be conserved and did not increase with female age, irrespective of the total number of eggs [12,47]. Given the allocation of the limited nutrition available to each offspring during the reproductive period, we found that the larger females mainly tend to improve their fitness by increasing their clutch frequency, which agrees with the findings of studies on the *Dermochelys coriacea* increasing their clutch frequency and enlarged clutch size to increase seasonal fecundity [9].

In one word, many effectors have mixed effects on the offspring size and number, especially when referring to old propagule. However, test turtles in previous studies (also our study included) clarify the relationship between maternal age and offspring size, and the number was relatively younger given their long life. Younger females grew more quickly than old females, which implied a trade-off between growth and reproduction, and this may lead to age effects tending to trump the size effects on the offspring size and number [48]. Therefore, a larger age range of the objects should be selected in future work, and more work needs to be conducted to validate and clarify the mechanism underlying it.

## 5. Conclusions

The number of offspring increased with the increasing maternal body size (plastron length), while there was no significant correlation between the maternal size and offspring size. During the reproductive season, larger females may increase their reproductive output by producing more, but not larger, offspring when compared with those of the smaller females. Our findings showed that the reproductive strategy of the cultured Asian yellow pond turtle is inconsistent with the optimal offspring size theory, enabling us to understand the evolution of the reproductive strategy in cultured reptiles and providing a basis for the efficient cultivation management of turtles. For instance, larger and older females should be selected for artificial breeding to produce more and larger offspring in actual cultivation.

## Figures and Tables

**Table 1:** The reproductive characteristics of 84 reproductive females.

Characteristics	Year			
	2013	2014	2015	2016
Number of offspring	287	291	350	331
Number of clutches	170	153	188	122
Percentages of egg-laying females (%)	97.62 (82/84)	96.43 (81/84)	100 (84/84)	92.86 (78/84)

**Table 2:** Mixed model (linear) and generalized linear model to evaluate maternal age (year) and body size on offspring size and the number of offspring.

Offspring Traits		Model			
Offspring size		Mixed model (linear)			
	Fixed effect	*df*	*ddf*	*F*	*p*
	Year (age)	3	186.82	30.17	**0.000**
	Plastron length	1	76.83	0.36	0.550
Number of offspring ^a^		Generalized linear model			
	Independent Variable	*df*		*χ* ^2^	*p*
	Year (age)	3		284.52	0.171
	Plastron length	1		272.28	**0.001**

^a^, the overall number of offspring per female in a year; *df*, degree of freedom; *ddf*, denominator degree of freedom; *χ*^2^, chi-square test; *p* < 0.05 means significant effects and shown in bold.

**Table 3:** The regression analyses among the female and offspring traits across four years.

Trait	Maternal Plastron Length			Offspring Body Mass			Clutch Size		
	*F*	*df*	*p*	*F*	*df*	*p*	*F*	*df*	*p*
Offspring body mass	0.03	1,82	0.876	/	/	/	**6.43**	**1,631**	**0.011**
Offspring number	**9.7**	**1,82**	**0.003**	**5.96 ^a^**	**1,82 ^a^**	**0.017 ^a^**	**590.6 ^a^**	**1,82 ^a^**	**<0.001 ^a^**
Clutch size	**9.63**	**1,82**	**0.003**	**6.43**	**1,631**	**0.011**	/	/	/
Clutch mass	**10.19**	**1,82**	**0.002**	**79.12**	**1,631**	**<0.001**	**8278**	**1,631**	**<0.001**

^a^, the results from the log_10_^Y^–log_10_^X^ relationship analysis; significant values (*p* < 0.05) shown in bold.

## Data Availability

The data presented in this study are available from the corresponding author upon request. The data are not publicly available due to institutional instructions.

## Supplementary Materials

The following supporting information can be downloaded at: https://www.mdpi.com/article/10.3390/ani13132219/s1, Figure S1: Maternal plastron length significantly influenced the mean clutch size (A), total clutch mass (B) and offspring number (C). Each point represents data from an individual mother.; Figure S2: Clutch mass increased with the mean offspring body mass (A). Mean offspring body mass increased significantly with increasing mean offspring number (B). Mean offspring body mass was positively correlated with clutch size (C). The offspring number was significantly influenced by the mean clutch size (D) after log10 transformation. Each point represents data from an individual clutch (A, C) and mother (B, D).
