# Supplementary material for: Reproductive Output Reveals the Maternal Effects on Offspring Size-Number Trade-Off in Cultured Asian Yellow Pond Turtle (Mauremys mutica)
Source: Animals (Basel). 2023 Jul 6;13(13):2219. doi: 10.3390/ani13132219 (PMC10340008; doi:10.3390/ani13132219)
Supplement: Supplementary file 1 [file animals-13-02219-s001.zip › animals-2412880-supplementary.pdf]

animals

MDPI

Supplementary Materials

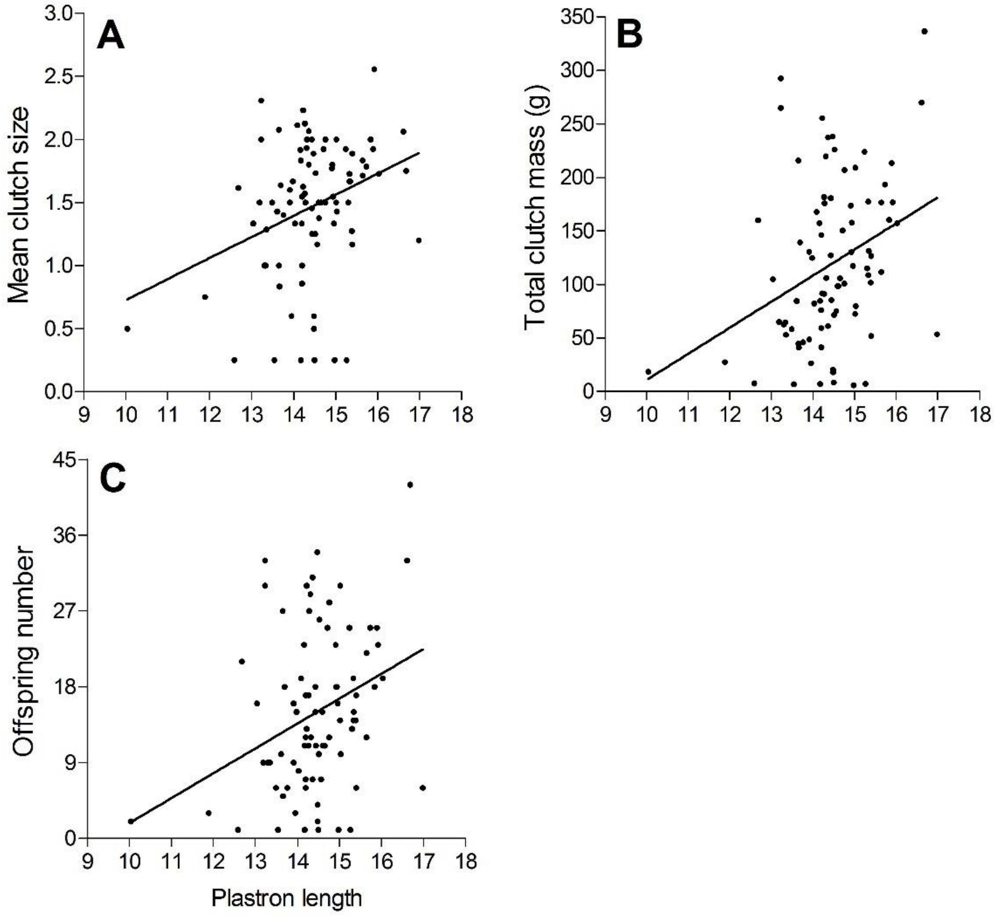

Figure S1. Maternal plastron length significantly influenced the mean clutch size (A), total clutch mass (B) and offspring number (C). Each point represents data from an individual mother.

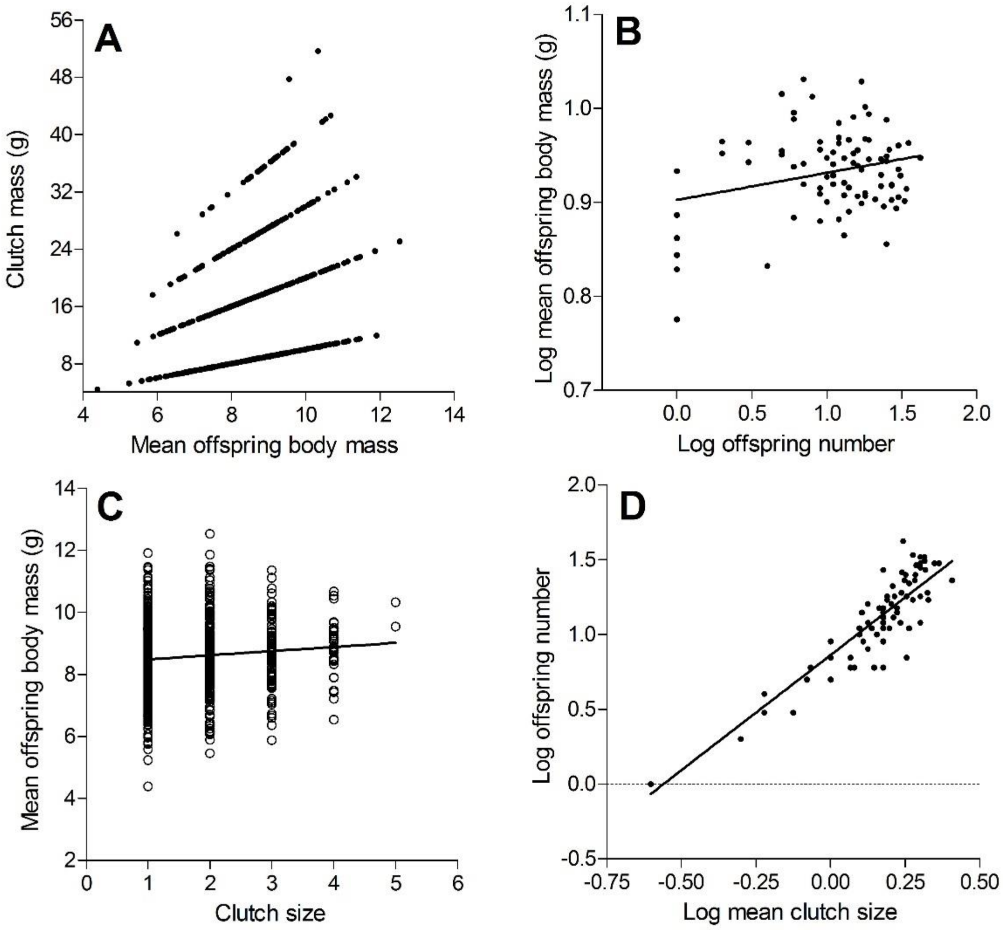

Figure S2. Clutch mass increased with the mean offspring body mass (A). Mean offspring body mass increased significantly with increasing mean offspring number (B). Mean offspring body mass was positively correlated with clutch size (C). The offspring number was significantly influenced by the mean clutch size (D) after log_{10} transformation. Each point represents data from an individual clutch (A, C) and mother (B, D).
